# Live birth after letrozole as an adjunct to follicle-stimulating hormone versus follicle-stimulating hormone alone for ovarian stimulation in in vitro fertilisation cycles—study protocol for a randomised controlled trial

**DOI:** 10.1186/s13063-022-06185-0

**Published:** 2022-04-01

**Authors:** Wei Guo, Hang Wun Raymond Li, Zi Yang, Lin Zeng, Rui Yang, Jie Qiao, Rong Li, Ernest Hung Yu Ng

**Affiliations:** 1 Centre for Reproductive Medicine, Department of Obstetrics and Gynecology, Peking University Third Hospital, No.49 North Huayuan Road, Haidian District, Beijing, 100191 China; 2 National Clinical Research Centre for Obstetrics and Gynecology, Beijing, 100191 China; 3 Key Laboratory of Assisted Reproduction (Peking University), Ministry of Education, Beijing, 100191 China; 4 Beijing Key Laboratory of Reproductive Endocrinology and Assisted Reproductive Technology, Beijing, 100191 China; 5 Department of Obstetrics and Gynaecology, The University of Hong Kong, Queen Mary Hospital, Pokfulam Road, Hong Kong, Special Administrative Region China; 6 Research Centre of Clinical Epidemiology, Peking University Third Hospital, 49 North Garden Rd., Haidian District, Beijing, 100191 China

**Keywords:** Letrozole, Estradiol, In vitro fertilisation, Fresh embryo transfer, Live birth rate, Randomised controlled trial

## Abstract

**Introduction:**

In vitro fertilisation (IVF) is an effective infertility treatment but the live birth rate remains unsatisfactory. Ovarian stimulation by follicle-stimulating hormone (FSH) is routinely used in IVF and the resulting high serum estradiol levels may impair oocyte/embryo quality and endometrial receptivity. Letrozole, an aromatase inhibitor, can reduce serum estradiol levels following ovarian stimulation. We aim to test the hypothesis that co-treatment with letrozole reduces supraphysiological serum estradiol levels and improves endometrial receptivity, leading to a higher live birth rate of IVF. We are conducting a randomised controlled trial (RCT) to evaluate whether letrozole as an adjunct to FSH in IVF is superior to FSH alone in the live birth rate of fresh embryo transfer.

**Methods/design:**

This is an open-label randomised controlled superiority trial being performed in two assisted reproduction centres in China. Infertile women who have antral follicle count (AFC) before ovarian stimulation or on day 5 of ovarian stimulation ≥15 are randomly allocated in a 1:1 ratio to receive either letrozole and FSH or FSH alone in a GnRH antagonist protocol. Recruited women follow the standard operating procedures of the two centres. The primary outcome is the live birth rate of the fresh embryo transfer. Stimulation parameters, maternal side effects and obstetric and perinatal complications are secondary outcomes. The planned sample size is 900, i.e. 450 per group.

**Discussion:**

The present study is the first multicentre randomised study to compare the live birth rate of the fresh embryo transfer following ovarian stimulation by letrozole and FSH versus FSH alone in women with anticipated high ovarian responses.

**Trial registration:**

ClinicalTrials.gov NCT02912988. Registered on September 23, 2016. This trial protocol is version 2.0.

## Background

Despite improvements in ovarian stimulation regimens and laboratory techniques, the live birth rate of in vitro fertilisation (IVF) remains unsatisfactory [1]. Ovarian stimulation is used in the great majority of IVF cycles and may lead to high serum estradiol (E2) levels with adverse effects on oocyte/embryo quality and endometrial receptivity [2–6].

High serum E2 levels have been implicated in reducing oocyte quality and increasing embryo aneuploidy rates [4–7]. The impact of supraphysiological E2 levels on endometrial receptivity includes disruption of endometrial gene expression [8], cytokine profiles [9] and histological markers of endometrial receptivity [10, 11]. High serum E2 level during ovarian stimulation is associated with an increased risk of small for gestational age and preeclampsia in singleton pregnancies after IVF [12].

Letrozole, a third-generation aromatase inhibitor, reduces the intraovarian aromatisation of androgens to oestrogens. It is now the first-line drug treatment for ovulation induction in women with polycystic ovary syndrome (PCOS) [13] and is routinely used as a co-treatment during ovarian stimulation in women with hormone-sensitive cancers such as breast cancer to reduce the risk of recurrence associated with high serum E2 levels during ovarian stimulation [14]. Peak serum E2 levels were significantly lower in the letrozole cohort [15–17].

Letrozole has been used for endometrial preparation in frozen embryo transfer (FET) in women both with anovulation caused by PCOS and with regular cycles. Retrospective studies [18–20] showed that the ongoing pregnancy rate or live birth rate of FET in letrozole-induced cycles was significantly higher than that in hormonal cycles of women with polycystic ovary syndrome or ovulation disorders. A large retrospective cohort study [21] reported the pregnancy outcomes of 110,722 FET cycles replacing a single embryo in letrozole, natural or hormonal cycles. The rates of clinical pregnancy, ongoing clinical pregnancy and live birth were significantly higher, while the rate of miscarriage was significantly lower in the letrozole group compared with the natural and hormone replacement therapy (HRT) groups.

The above studies indicate that adding letrozole to ovarian stimulation may improve endometrial receptivity by reducing supraphysiological serum E2 levels and have a direct effect on endometrial receptivity. By combining sufficient FSH stimulation to produce adequate numbers of oocytes, with a simple oral adjuvant therapy aimed at preventing excessive estradiol levels, the resultant stimulation protocol may provide a ‘best of both worlds’ solution, in which adequate numbers of oocytes are obtained, but not at the cost of detrimental impacts on oocyte, embryo or endometrial quality.

The aim of this multicentre randomised controlled trial is to test the hypothesis that co-treatment with letrozole leads to a higher live birth rate of fresh embryo transfer.

### Study setting

This is an open-label randomised controlled superiority trial being performed in the Department of Obstetrics & Gynaecology, The University of Hong Kong, and the Centre for Reproductive Medicine, Department of Obstetrics and Gynecology, Peking University Third Hospital. Written informed consent was obtained from all participants before participation in the study.

## Methods/design

### Study design

Eligible women will be given information about the study during their first consultation. All recruited women provided written informed consent before participation. The trial was approved by the institutional review board of the University of Hong Kong/Hospital Authority Hong Kong West Cluster (UW16-014) and the Ethics Committee of Peking University Third Hospital (2016sz-066). The study was registered on Clinicaltrials.gov (NCT02912988).

**Fig. 1:**
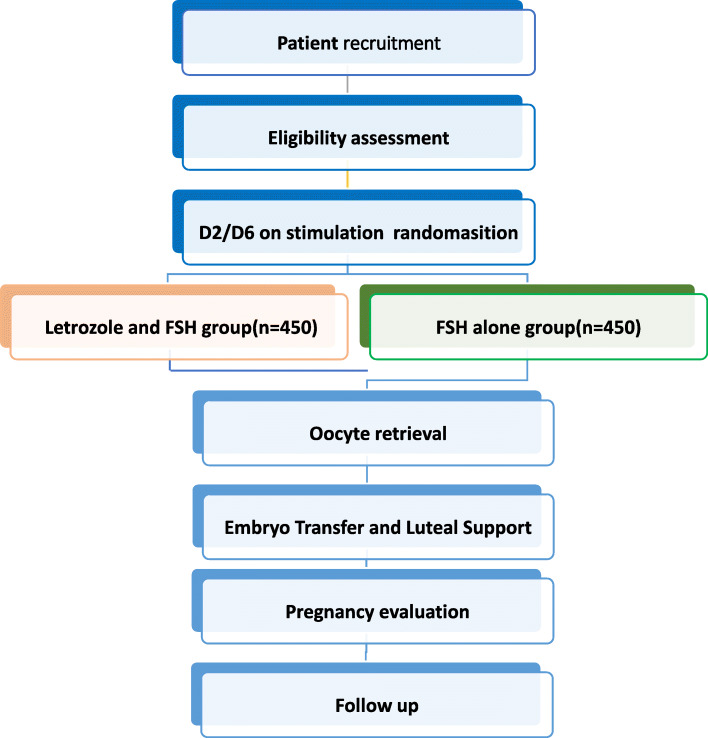
Flowchart of clinical RCT study: letrozole + FSH vs FSH alone for ovarian stimulation in IVF

The enrollment, interventions and evaluation during the study process are shown in Table 1. Figure 1 indicates a flowchart showing registration, allocation, intervention and follow-up of participants.

**Table 1:** Schedule of enrollment, interventions and assessments

	Study period							
	Enrollment	Allocation		Post-allocation				Close-out
Content	Screening and baseline assessment	Ovarian stimulation and randomisation	Oocyte retrieval	Assessment of embryo	Embryo transfer	Evaluation of pregnancy	Follow-up of pregnancy	
Time point	T0 −1 month	T1 0 month	T2 8–12 days	T3 1–3–5 days after retrieval	T4 2–5 days	T5 1 month	T6 3–10 months	T7 12 months
Enrollment								
Eligibility screen	×	×						
Informed consent		×						
Allocation		×						
Interventions								
Letrozole and FSH		×						
FSH alone		×						
Assessments								
Baseline data	×							
Laboratory tests	×	×	×			×	×	×
Fertilisation check				×				
Embryo quality				×	×			
Pregnancy tests						×		
Pregnancy outcomes							×	×
Foetus information							×	×
Neonate information							×	×
Safety assessment		×	×		×	×	×	×

### Recruitment

Infertile women undergoing IVF treatment at the Department of Obstetrics & Gynaecology, the University of Hong Kong, and the Centre for Reproductive Medicine, Department of Obstetrics and Gynecology, Peking University Third Hospital, will be screened for eligibility. Eligible women will be counselled about the trial on the first day attending the centres for ovarian stimulation. Written informed consent will be obtained before joining the trial. Participants can decline to join the study at any time without any consequences for their clinical treatments.

### Eligibility criteria

#### Inclusion criteria

- Women less than 42 years of age
- Having a medical indication for IVF treatment
- Total antral follicle count (AFC) on pelvic scanning before ovarian stimulation or on day 5 of stimulation ≥15
- Written informed consent obtained

#### Exclusion criteria

- Women using donor oocytes
- Women undergoing preimplantation genetic testing
- Women with abnormal uterine cavity shown on hysterosalpingogram or saline infusion sonogram
- Women with hydrosalpinges shown on scanning and not corrected
- Having previously documented poor response, i.e. ≤3 oocytes to ovarian stimulation using at least FSH 225 IU daily

### Randomisation and allocation concealment

Recruited women will be randomly allocated in a 1:1 ratio to the letrozole group or the control group according to a computer-generated randomisation list put in opaque sealed envelopes which are open consecutively for eligible participants after screening. Both investigators and participants are aware of the allocation of the intervention. The biostatistician performing the data analysis will be blinded to the group allocation.

### Interventions

A gonadotropin-releasing hormone (GnRH) antagonist regimen is used. Human menopausal gonadotropin (HMG; Menopur, Ferring Pharmaceuticals, Saint-Preix, Switzerland) or recombinant human follicle-stimulating hormone (FSH; Gonal-F, Merck Serono, Geneva, Switzerland) is administered at a dose of 150 to 225 IU per day based on body mass index and previous ovarian response on day 2–4 of the menstrual cycle, at the discretion of the physicians. Transvaginal scanning, serum luteinizing hormone (LH), oestrogen (E2) and progesterone (P4) will be performed regularly to monitor ovarian response. HMG or FSH doses will be adjusted according to ovarian responses.

GnRH antagonist 0.25 mg (Cetrotide, Merck Serono, Darmstadt, Germany, or Orgalutran, Merck Sharp & Dohme Ltd.) is started from day 5 of stimulation until the day of the trigger. When three or more follicles reach a diameter ≥ 17 mm, 250μg of rhCG (Ovidrel; Serono, Aubonne, Germany) or triptorelin 0.2 mg (Diphereline, Ipsen Beaufour) is to be given for final maturation. Oocyte retrieval is performed around 36 h after the trigger.

In the letrozole group, letrozole (Letroz, Sun Pharmaceuticals, Mumbai, India) 2.5 mg daily is started from day 5 of stimulation until the day before the trigger. The control group will not receive letrozole.

When the serum estradiol level on the day of hCG administration or before the day was ≥20000 pmol/L, 0.2 mg of triptorelin (Ferring International Center, Saint-Prex, Switzerland) will be given for agonist trigger. When the serum estradiol level on the day of hCG administration or before the day was 15,000–20,000 pmol/L, 0.2 mg of triptorelin (Ferring International Center, Saint-Prex, Switzerland) and 2000 IU of hCG (Livzon, Zhuhai, China) or 125 mg of recombinant hCG (Ovidrel; Merck Serono) will be given.

The freeze-all strategy to prevent ovarian hyperstimulation syndrome (OHSS) is advised in women with (1) E2 on HCG day ≥15,000 pmol/L, (2) retrieved oocyte number ≥ 20, and (3) E2 on HCG day ≥10,000 pmol/L and retrieved oocyte number ≥ 15.

### Assessment of fertilisation and embryo culture

Oocytes are cultured in Human Tubal Fluid medium and incubated in a humidified 37 °C incubator with 5% CO2, after oocyte retrieval immediately. The fertilisation method will be selected according to the parameters of the semen analysis.

Fertilisation will be considered normal when two pronuclei are present between 16 and 18 h after conventional insemination or intracytoplasmic sperm injection (ICSI). All zygotes will be cultured in a cleavage medium (G-1plus, Vitrolife, USA) for further 48–52 h after fertilisation. Cleavage stage embryos will be assessed according to the developmental stage and degree of cytoplasmic fragmentation.

### Embryo transfer and luteal support

Fresh embryo transfer or elective freezing of all embryos will be decided by physicians according to the conditions of women. All embryos will be cryopreserved without fresh transfer when the women are at risk of OHSS, have elevated serum progesterone level on the trigger day (> 1.5 ng/L or > 5 nmol/L) or had endometrial polyps or fluid in the uterine cavity. To reduce the risk of multiple pregnancies, up to two embryos will be transferred on day 2/3 and one blastocyst will be replaced on day 5. Excess cleavage stage embryos will be vitrified or further cultured to the blastocyst stage while excess blastocyst will be vitrified accordingly.

In women with fresh embryo transfer, the luteal phase is supported with vaginal progesterone gel (Crinone, Merck Serono) at a dose of 90 mg daily or oral progesterone (Duphaston, Abbott) at a dose of 10 mg three times daily from the day of oocyte retrieval until the day of serum or urine hCG test.

### Follow-up

Transvaginal ultrasonography will be performed 2 weeks after a positive serum or urine pregnancy test to confirm intrauterine pregnancy. Scanning will be repeated later to confirm ongoing pregnancy, which is defined as a viable pregnancy with a foetal heartbeat at 10–12 weeks. Information regarding the outcome of the pregnancy and obstetrical and perinatal complications will be obtained through a review of obstetrical medical records and neonatal medical records. The live birth is defined by the live foetus in the uterine after 20 weeks of gestation.

We will collect the following information within 6 weeks after delivery, including antenatal information (pregnancy complications and foetal information), delivery information (gestational age, mode of delivery, placental weight and birth complications) and newborn information (foetal sex, birth weight and birth defects).

### Measurement of outcomes

#### Primary outcome

The primary outcome is the live birth rate after 20 weeks of gestation following the fresh transfer.

#### Secondary outcomes

Secondary outcomes include pregnancy outcomes, maternal safety and obstetric and perinatal complications. Detailed information is provided in Table 2. Maternal safety is an important part of the secondary outcomes. The incidence of OHSS is the key indicator of maternal safety and is classified as mild, moderate or severe according to the RCOG guideline [22, 23]

**Table 2:** Secondary outcomes and related definition

Secondary outcomes	Definition
Clinical and laboratory outcomes	
Total amount of FSH	Total amount of FSH used for ovarian stimulation
Number of follicles > 12 mm	The number of follicles > 12 mm measured by transvaginal ultrasound on the day of the trigger or the day before
Number of oocytes obtained	Number of oocytes obtained during oocyte retrieval
Number of embryos obtained	Number and quality of embryos obtained
Proportion of good-quality embryos	Embryos with 4 cells and < 25% fragmentation on day 2 or with ≥6 cells and ≤ 10% fragmentation on day 3, the proportion of oocytes resulting in top quality day 2 (or day 3) embryos according to validated morphological criteria
Oocyte fertilisation rate	The number of zygotes with 2PN over the number of mature oocytes for ICSI or over the number of oocytes for conventional insemination
Endometrial thickness	Endometrial thickness measured by transvaginal ultrasound on the day of the trigger or the day before
Serum E2 level	Serum E2 level on the day of the trigger or the day before
Serum P level	Serum P level on the day of the trigger or the day before
Serum testosterone level	Serum testosterone level on the day of the trigger or the day before
Follicular fluid E2 level	E2 level in the follicular fluid
Follicular fluid testosterone level	Testosterone level in the follicular fluid
Follicular fluid inhibin B level	Inhibin B level in the follicular fluid
Follicular fluid AMH level	AMH level in the follicular fluid
Pregnancy outcomes	
Miscarriage	Spontaneous loss of an intrauterine pregnancy before 20 completed weeks of gestational age
Clinical pregnancy	Presence of one or more intrauterine gestational sacs at 6 weeks of gestation
Ongoing pregnancy	Presence of one or more gestational sacs and foetal heartbeat after 12 weeks of gestation
Maternal safety outcomes	
OHSS	It is classified as mild, moderate or severe according to the degree of abdominal distention, ovarian enlargement and respiratory haemodynamic, and metabolic complications. Diagnosed by ultrasound, blood testing and physical examination according to the RCOG Guideline
Ectopic pregnancy	A pregnancy outside the uterine cavity, diagnosed by ultrasound, surgical visualisation or histopathology
Obstetric and perinatal complications	
Hypertensive disorders of pregnancy	Including pregnancy-induced hypertension, preeclampsia and eclampsia
Antepartum haemorrhage	Including placenta previa, placenta accreta and unexplained
Multiple pregnancies	Pregnancy with more than one foetus
Birth weight	Including low birth weight (weight < 2500 g at birth), very low birth weight (< 1500 g at birth), high birth weight (> 4000 g at birth) and very high birth weight (> 4500 g at birth)
Small for gestational age	Birth weight less than the 10th centile for the sex-specific birth weight for a given gestational age reference
Preterm delivery	Birth of a foetus delivered after 28 and before 37 completed weeks of gestational age
Congenital anomaly	Structural or functional disorders that occur during intrauterine life and can be identified prenatally, at birth or later in life, including trisomy 21 syndrome, neural tube defect, congenital heart disease, cleft lip, excessive numbers of fingers or toes and hydrocephalus
Perinatal mortality	Foetal or neonatal death occurring during late pregnancy (at 28 completed weeks of gestational age and later), during childbirth, or up to seven completed days after birth

### Safety reporting

Adverse events (AE) are defined as any undesirable experience occurring to a subject during the trial. A serious adverse event (SAE) is any untoward medical event that results in death, is life-threatening (at the time of the event), requires hospitalisation or prolongation of existing inpatients’ hospitalisation, results in persistent or significant disability or incapacity, is a congenital anomaly or birth defect and is a new event of the trial likely to affect the safety of the subjects, such as an unexpected outcome of an adverse reaction.

SAE in this study includes moderate/severe OHSS, intraperitoneal haemorrhage or ovarian torsion after oocyte retrieval, ectopic pregnancy, severe preeclampsia, pregnancy complications leading to hospitalisation, stillbirth, birth defects and other serious medical events judged by researchers to meet the criteria of SAE.

### Statistical analysis

#### Sample size calculation

The primary hypothesis of this trial is that the letrozole group will increase the live birth rate when compared with the control group. This treatment will be considered effective if the proportion of women having a live birth is increased by 10% in absolute terms. With an 80% power and a one-sided 2.5% level of statistical significance, we will need to recruit 752 women (376 in each arm) to show an absolute difference of 10% from 35 to 45% between control and letrozole groups. Taking consideration of the dropout rate as 20% (such as cancellation of fresh embryo transfer because of high risk of OHSS), each group will include 450 women (a total of 900 women).

### Statistical analysis

The result will be analysed according to the intention-to-treat (ITT) principle. Per-protocol (PP) analysis will also be conducted as a sensitivity analysis. Baseline characteristics will be described by descriptive analysis and the balance among groups or subgroups will be assessed by analysis for different kinds of data. For continuous variables, the normality distribution will be estimated by using frequency histograms and the Kolmogorov-Smirnov test initially. If the continuous variables are normally distributed, they will be presented as means with standard deviations (SDs). If the continuous variables are non-normally distributed, their medians and interquartile ranges (IQRs) will be reported. For categorical variables, we will present the proportion between each group. In addition, the recruitment number, those participants lost to follow-up, protocol violations and other relevant data will also be reported. A comparison between groups will be performed using the independent sample *t*-test, Mann-Whitney *U* test for continuous variables or Pearson chi-square test/Fisher’s exact test for categorical variables as appropriate. The primary outcome will be compared using Pearson’s chi-square test or Fisher’s exact test as appropriate. Categorical secondary outcomes will be compared between two groups using a similar approach as the primary outcome. Student’s *t*-test or Wilcoxon test will be used as appropriate for continuous secondary outcomes, such as birth weight, etc. The relative risks (RR) and absolute rate differences (ARD) and their 95% confidence interval (CI) between the two groups will be calculated. And the 99% CI of the ARDs will be used to evaluate if the letrozole group is superior to the control group. Multiple variable logistic regression models will be used to assess the treatment effect adjusting for other potential confounding variables that are unbalanced in the baseline.

Missing data will be treated as missing at random and will be imputed using the last observation carried forward (LOCF) method. For the missing values, a sensitivity analysis will be done under the hypothesis of the worst and the best outcomes for each missing individual. Therefore, all secondary outcomes will be considered exploratory. All statistical analyses will be done using the statistical package SPSS (version 26.0, released 2019, IBM corp., Armonk, NY, USA). Statistical significance is defined as *p* < 0.05 with two-sided testing.

## Discussion

Letrozole is usually used for ovulation induction in anovulatory women and ovarian stimulation and intrauterine insemination for treatment of unexplained infertility. It is now routinely used in women with breast cancer undergoing fertility treatment in order to prevent recurrent breast cancer as a result of supraphysiological estradiol level following ovarian stimulation [14]. Its use in poor responders and normal responders in IVF may reduce the total gonadotropin dose required for ovarian stimulation without increasing the pregnancy rate [24–26]. The hypothesis of the present study is that adding letrozole to gonadotropin in the ovarian stimulation protocol in high responder patients can improve the live birth rate of women with anticipated high ovarian response in IVF. The present study is the first randomised study which provides high-quality evidence about the efficacy of co-administration of letrozole in high ovarian responders and IVF cycles and may open new insights into the clinical management of this group of women.

The strengths of the present study include randomisation, a large adequate sample size to show a 10% absolute difference in the live birth rate between the intervention and control groups and the live birth rate as the primary outcome, which is the most important clinical outcome in fertility treatment studies. Most women prefer to choose embryo transfer in the fresh cycle instead of transfer in frozen cycles because of time constrain, economic reasons and psychological factors, so the use of letrozole during ovarian stimulation for IVF producing a good number of oocytes with physiological levels of estradiol may reduce the adverse impact on endometrial receptivity and increase the success rate of the IVF treatment. To increase the external validity of this trial, we did not limit embryo transfer to day 3 or day 5 only and the results of day 3 and day 5 transfer will be separately analysed. The timing of embryo transfer is unlikely to influence the results because of randomisation in the study.

The limitation is the lack of cumulative live birth rate of the stimulated IVF cycle.

### Trial status and peer review

The first participant was recruited in March 2018, and it is anticipated the recruitment will end by the end of April 2022. The follow-up is ongoing and the expected data collection will be completed in February 2023. This trial protocol is version 2.0, 20/11/2017. At the time of the manuscript preparation, we have recruited 890 women and the recruitment is currently ongoing. This study is externally peer-reviewed and does not receive any external funding.

## Data Availability

The datasets used during the current study are available from the corresponding author on reasonable request.

## Abbreviations

AE: Adverse events
AFC: Antral follicle count
E2: Estradiol
FET: Frozen embryo transfer
FSH: Follicle-stimulating hormone
GnRH: Gonadotropin-releasing hormone
hCG: Human chorionic gonadotropin
HMG: Human menopausal gonadotropin
HRT: Hormone replacement therapy
IVF: In vitro fertilisation
ICSI: Intracytoplasmic sperm injection
LH: Luteinizing hormone
OHSS: Ovarian hyperstimulation syndrome
P4: Progesterone
PCOS: Polycystic ovary syndrome
RCT: Randomised controlled trial
SAE: Serious adverse event
